# Metformin and ICG-001 Act Synergistically to Abrogate Cancer Stem Cells-Mediated Chemoresistance in Colorectal Cancer by Promoting Apoptosis and Autophagy

**DOI:** 10.3390/cancers14051281

**Published:** 2022-03-02

**Authors:** Souvick Roy, Yinghui Zhao, Yate-Ching Yuan, Ajay Goel

**Affiliations:** 1 Department of Molecular Diagnostics and Experimental Therapeutics, Beckman Research Institute of City of Hope, Monrovia, CA 91016, USA; soroy@coh.org (S.R.); yinghuizhao@mail.sdu.edu.cn (Y.Z.); 2 Department of Clinical Laboratory, The Second Hospital, Cheeloo College of Medicine, Shandong University, Jinan 250033, China; 3 Bioinformatics Core Facility, City of Hope National Medical Center, Duarte, CA 91010, USA; yyuan@coh.org; 4 Department of Medical Oncology, City of Hope National Medical Center, Duarte, CA 91010, USA; 5 City of Hope Comprehensive Cancer Center, Duarte, CA 91010, USA

**Keywords:** colorectal cancer, 5FU-resistance, cancer stem-like cells, metformin, ICG-001

## Abstract

**Simple Summary:**

Colorectal cancer (CRC) is one of the most frequently diagnosed and lethal malignancies. The majority of CRC patients experience disease relapse after the primary curative treatment strategy of surgery followed by 5FU-based chemotherapy. The presence of cancer stem-like cells (CSCs) is considered to be one of the contributing factors to therapy resistance and disease relapse in CRC. Previous studies implicated the role of the Wnt signaling pathway in the maintenance of the CSC phenotype. Therefore, in this study we explored a novel therapeutic strategy using metformin along with ICG-001, a Wnt signaling inhibitor, to abrogate CSC-mediated chemoresistance in CRC. We observed that metformin and ICG-001 abrogate stemness in a synergistic manner by promoting autophagy and apoptosis in 5FU-resistant CRC cells as well as in CRC patient-derived tumor organoids. Hence, metformin and ICG-001 can be used as part of a therapeutic strategy to overcome 5FU-mediated therapeutic resistance in CRC.

**Abstract:**

Colorectal cancer (CRC) remains the third most frequently diagnosed cancer in the United States. The current treatment regimens for CRC include surgery followed by 5FU-based chemotherapy. Cancer stem-like cells (CSCs) have been implicated in 5FU-mediated chemoresistance, which leads to poor prognosis. In this study, we used metformin along with ICG-001, a Wnt signaling inhibitor, to abrogate CSC-mediated chemoresistance in CRC. We observed that 5FU-resistant (5FUR) CRC cells exhibited increased expression of CSC markers and enhanced spheroid formation. Genome-wide transcriptomic profiling analysis revealed that Wnt signaling, colorectal cancer metastasis signaling, etc., were enriched in 5FUR CRC cells. Accordingly, selective targeting of Wnt signaling using ICG-001 along with metformin abrogated CSC-mediated chemoresistance by decreasing the expression of CSC markers and promoting autophagy and apoptosis in a synergistic manner. We also observed that metformin and ICG-001 exhibited anti-tumor activity in CRC patient-derived tumor organoids. In conclusion, our study highlights that metformin and ICG-001 act synergistically and can be used as part of a therapeutic strategy to overcome 5FU-mediated therapeutic resistance in CRC.

## 1. Introduction

Colorectal cancer (CRC) is the third most frequently diagnosed cancer worldwide; the American Cancer Society estimated approximately 147,950 new cases of CRC in 2020 in the United States alone [1]. Several key factors, including poor dietary habits, smoking, alcohol consumption, genetic predisposition, obesity, diabetes mellitus, and sedentary lifestyles, result in a significantly increased risk for developing CRC [2]. Current treatment modalities for patients with CRC include surgery, which is often followed with adjuvant chemotherapy, especially in patients with stage II and III disease [3]. A majority of patients with advanced CRC are generally treated with the chemotherapeutic drug, 5-fluorouracil (5FU), either given alone or in combination with oxaliplatin and other molecularly targeted drugs [4]. While such treatment regimens are effective in improving disease outcomes, their clinical usefulness is often hampered due to the considerable toxicity associated with these treatments, which often cause severe nausea, vomiting, and weight loss, and increase the risk of infectious complications due to immunosuppression [5,6]. Furthermore, their therapeutic efficacy is limited due to the emergence of chemotherapeutic drug resistance [7,8]. While different chemotherapeutic regimens possess varying therapeutic responses, each regimen is often accompanied with significant adverse effects, therapeutic resistance, and disease relapse, collectively highlighting the need to develop improved therapeutic modalities to treat this malignancy.

Intriguingly, it has been reported that the risk of CRC increases up to three times with diabetes mellitus, predominately type 2 diabetes mellitus (T2DM) [9]. T2DM is a chronic metabolic disorder associated with high mortality and morbidity. Patients with T2DM have an increased risk of various malignancies due to other underlying conditions, including hyperinsulinemia, hyperglycemia, and inflammation [10]. Furthermore, T2DM has been shown to increase CRC aggressiveness and mortality [9]. Metformin (1,1-dimethylbiguanide), a product of French lilac (*Galega officinalis*), is an oral biguanide and a hypoglycemic agent that is prescribed to over 120 million patients with gestational diabetes, T2DM, non-alcoholic fatty liver disease, premature puberty, and polycystic ovarian syndrome (PCOS) worldwide [2]. Previous studies based on pancreatic, colorectal, and ovarian cancers reported that metformin treatment significantly decreased cancer incidence rates [11]. The molecular mechanism(s) of action for metformin’s activity involve the AMPK/mTOR signaling pathway, wherein metformin treatment mediates LKB1-dependent activation of AMPK, which sequentially inactivates mTOR [12,13,14]. In addition, metformin treatment also associates with p53 activation, which promotes autophagy and decreased protein synthesis [15,16,17]. Metformin inhibits cell proliferation and promotes cell cycle arrest in breast cancer [18]. Furthermore, metformin treatment selectively targets CD133+ CSC populations and affects cell proliferation, apoptosis, and cell cycle arrest in CRC cells [19].

CSCs promote tumorigenesis and metastasis and mediate resistance to chemo- and radiotherapy, which eventually results in disease relapse in different cancers [20,21]. Colorectal CSCs are often identified through the expression of cell surface markers, such as CD44, CD133, CD166, Lgr5, ALDH1, and EpCAM [22,23]. In a previous study from our group, we observed that CD44v6 represented a CSC population with increased resistance to chemotherapeutic agents, and its high expression was frequently associated with poor overall survival (OS) and disease-free survival (DFS) in CRC patients [20]. In this context, the Wnt signaling pathway is implicated in the maintenance of the CSC phenotype in different solid tumors [21,24]. In a previous study, it was observed that 5FU treatment promoted stemness of CRC by activating Wnt/β-catenin signaling in a p53-dependent manner [25], hence highlighting that targeting the Wnt/β-catenin signaling pathway can facilitate the inhibition of the CSC phenotype in various cancers, including CRC.

In view of the anti-cancer effects of metformin and the key role that Wnt/β-catenin signaling plays in CRC, we hypothesized that a combined treatment of metformin and the Wnt pathway inhibitor ICG-001 might offer a superior abrogation of CSC-mediated chemoresistance in CRC. Accordingly, following a systemic investigation in a parental and 5FU-resistant cell line model along with patient-derived organoids, we observed that metformin and ICG-001 act synergistically through the modulation of autophagy and apoptosis. The clinical impact of our findings could form the basis of using metformin as an adjuvant treatment along with conventional chemotherapy in CRC—an approach which may lead to increased therapeutic efficacy and reduced gastrointestinal toxicity for the management of patients with this fatal malignancy.

## 2. Materials and Methods

### 2.1. Drugs, Antibodies, and Primers

The 5-fluorouracil, metformin, and ICG-001 used were purchased from Sigma-Aldrich (St. Louis, MO, USA) and Selleckchem (Houston, TX, USA) respectively. The antibodies, such as anti-PARP, anti-cleaved PARP, anti-caspase 3, anti-cleaved caspase 3, anti-LC3B, anti-ATG9A, anti-CD44, anti-β-catenin, anti-OCT4, anti-β-actin, anti-p-AMPK, and anti-AMPK were procured from Cell Signaling Technology (Danvers, MA, USA). APC conjugated anti-CD44 was procured from BioLegend (San Diego, CA, USA). All the primers were procured from Integrated DNA Technologies (San Diego, CA, USA).

### 2.2. Cell Culture

The human CRC cell lines, HCT116, SW480, HT29, SW620, and RKO, were obtained from the American Type Culture Collection (ATCC, Manassas, VA, USA) and the normal colonic epithelial cell line, NCM460, was obtained from the INCELL Corporation (San Antonio, TX, USA). The 5FU-resistant (5FUR) cell lines (HCT116-5FUR and SW480 5FUR) were established by continuous culturing of cells with increasing doses of 5FU for more than 9 months, as described previously [8,26,27,28,29,30]. CRC cells were cultured as monolayers, as reported previously [27,29].

### 2.3. Cell Viability Assay

Cell viability of parental and 5FU-resistant CRC cells were determined after treatment with different concentrations of metformin (1–20 mM) and ICG-001 (1–20 µM), alone or in combination, for 48 h using the Cell Counting Kit-8 (CCK-8) (Dojindo, Kumamoto, Japan), as reported previously [29]. The spheroids were dissociated through enzymatic treatment and grown as monolayers and treated with increasing concentrations of 5FU (5–200 µM) for 48 h. Following the CCK-8 assay, the IC_50_ values for metformin, ICG-001, and 5FU were determined using GraphPad Prism 8 software (La Jolla, CA, USA).

### 2.4. Isobologram Analysis 

The IC_50_ values obtained from the CCK-8 assay for metformin and ICG-001 were used for isobologram analysis. In this analysis, cells were treated with metformin and ICG-001 at a constant ratio based on several concentrations above and below the IC_50_ values of each drug. The combination index (CI) was calculated according to the classic isobologram equation by Chou et al. using Compusyn Software [31]. The ‘combination index’ (CI) was considered to depict synergism (CI < 1), additive effect (CI = 1), and antagonism (CI > 1) for metformin and ICG-001.

### 2.5. Clonogenic Cell Survival Assay

Colony formation capacities of parental and 5FU-resistant CRC cells were determined by clonogenic cell survival assays, as reported previously [21,32]. The cells were thereafter treated with different concentrations of metformin and ICG-001, either alone or in combination, for 48 h. The data are represented as number of colonies formed per 500 cells seeded.

### 2.6. Apoptosis and Cell Cycle Analysis

The percentage of apoptotic cells and cell cycle distribution were measured using a Muse Annexin V and Dead Cell Assay Kit (Millipore, Chicago, IL, USA) and Muse Cell Cycle Assay kit (Millipore) on a Muse Cell Analyzer (Millipore), according to the manufacturer’s instructions, after treatment with metformin and ICG-001, individually or in combination, in parental and 5FUR CRC cells.

### 2.7. Spheroid Formation Assay

Parental and 5FU-resistant CRC cells were subjected to spheroid formation assay, as described previously [21]. For these experiments, 500 cells/well were seeded in ultra-low attachment plates in serum-free DMEM-F12 medium (STEMCELL Technologies, Vancouver, BC, Canada) which was supplemented with 10 ng/mL epidermal growth factor (GIBCO), 10 ng/mL basic fibroblast growth factor (GIBCO), and 1% B27 supplement (GIBCO). After 3–4 days, when sphere-like structures were visible, the spheroids were treated with metformin and ICG-001, either alone or in combination, for 48 h. The images of spheroids for each treatment group were captured using a Zeiss AxioCam 702 sCMOS Mono at 10× magnification.

### 2.8. Flow Cytometric Analysis of CD44

Flow cytometric analysis was carried out to determine the percentage of CD44-positive cells in the population of parental, 5FUR cells and their respective spheroidal counterparts. First, cells were harvested and resuspended in dilution buffer (2% FBS in PBS). Next, APC conjugated CD44 primary antibody (1:400 dilution) was added to each sample and incubated for 1 h at 4 °C. After incubation, cells were washed 3 times with ice-cold PBS and resuspended in dilution buffer. Flow cytometric analysis was performed using Attune Nxt (Invitrogen, Carlsbad, CA, USA).

### 2.9. Wound Healing Assay

Wound healing assays were performed for the CRC cells after treatment with metformin and ICG-001, alone or in combination, as per the protocol reported earlier [32]. The cells were treated with metformin and ICG-001, either alone or in combination, and allowed to grow for 24 h. The images of the wounds were captured at 0 and 24 h time intervals using a Zeiss AxioCam 702 sCMOS Mono at 10× magnification. The percentage wound closure was calculated using Image J software ver. 1.52 (NIH, Bethesda, MD, USA).

### 2.10. Invasion Assay

The invasion assays were performed for CRC cells after treatment with metformin and ICG-001, alone or in combination, using a Matrigel invasion chamber (Corning, Tehama County, CA, USA) with an 8.0 µm PET membrane, as per the manufacturer’s instructions. The invaded cells were fixed with methanol and stained with 0.1% crystal violet (Acros Organics, Gujarat, India). The images were captured using a Zeiss AxioCam 702 sCMOS Mono at 10× magnification.

### 2.11. CRC Patient-Derived Tumor Organoids

Human primary CRC tissues were obtained from patients with CRC at the Baylor University Medical Center, Dallas, TX. All experiments were approved by the institutional review board of the institution. Written informed consent was obtained from all patients, in accordance with Declaration of Helsinki. The clinical details of the CRC patients used for organoid generation are provided in Supplementary Table S1. The CRC organoids were cultured as reported previously [23,27]. For treatment with metformin and ICG-001, either alone or in combination, the organoids were randomly assigned into four groups, and appropriate concentrations of metformin (3 mM), ICG-001 (5 µM), or their combination with DMSO as vehicle control were added to the culture medium of each group and cultured for 10 days. The images of organoids for each treatment group were captured after 10 days using a Zeiss AxioCam 702 sCMOS Mono at 10× magnification. The organoids were harvested by Gentle Cell Dissociation Reagent (STEMCELL Technologies) and were used further for RNA isolation and protein lysate preparation.

### 2.12. RNA Isolation, cDNA Synthesis, and Gene Expression Profiling by qPCR

CRC cells and patient-derived tumor organoids were used for total RNA extraction using QIAZOL reagent (Qiagen, Hilden, Germany) and then reverse-transcribed into cDNA using a High-Capacity cDNA Reverse Transcription Kit (Applied Biosystems, Foster City, CA, USA), as reported earlier [29,30]. Quantitative real-time PCR (qRT-PCR) analysis was performed for β-catenin, CSC markers (OCT4, CD44), apoptotic marker (caspase 3), and autophagy marker (LC3B) using a SensiFAST™ SYBR^®^ Lo-ROX Kit (Bioline; London, UK). The cycling condition includes 95 °C for 20 s, followed by 40 cycles of 95 °C for 1 s and 60 °C for 20 s, followed by melt curve analysis using a Quantstudio 7 Flex Real Time-PCR system. β-actin was used as a housekeeping gene and mRNA fold change was calculated using the 2^−ΔΔCT^ method [33]. The primer sequences are provided in Supplementary Table S2.

### 2.13. Western Blot Analysis

CRC cells and patient-derived organoids were used for extraction of protein followed by Western blot (WB) analysis, as reported previously [29,32]. Briefly, the cells and dissociated organoids were lysed using Pierce RIPA lysis buffer (Thermo Fisher Scientific, Waltham, MA, USA) supplemented with protease inhibitor cocktails (Thermo Fisher Scientific) to obtain protein lysates from total cells. The nuclear and cytoplasmic protein extraction was performed using NE-PER Nuclear and Cytoplasmic Extraction Reagents as per the manufacturer’s protocol (Thermo Fisher Scientific). Protein lysates were separated on 10–12% SDS-PAGE and transferred onto PVDF membranes. The membranes were blocked with 5% bovine serum albumin (Sigma-Aldrich) in Tris buffer saline (Bio-Rad, Hercules, CA, USA) with 0.1% Tween-20 (Sigma-Aldrich) for 1 h at room temperature (RT) and then probed with the primary antibody (1:2000) at 4 °C overnight. The membranes were washed with 0.1% TBST and probed with a secondary antibody (1:4000) for 1 h at RT. The signals were detected by using ChemiDoc-MP Imaging system (ver 5.2.1, Bio-Rad), using HRP-based chemiluminescence kit (ThermoFisher Scientific). The band intensities were quantified using the Image J software ver. 1.52 (NIH, Bethesda, MD, USA). The relative protein expression was measured with respect to control after normalizing with loading control (β-actin). LC3B-II/LC3-I ratios were measured after normalizing the band intensity of the respective protein markers with β-actin. The original blots can be found in Figure S2.

### 2.14. Genome-Wide Transcriptomic Profiling and Pathway Enrichment Analysis

Total RNAs from parental and 5FU-resistant CRC cells were subjected to genome-wide transcriptomic analysis [27]. Briefly, next generation sequencing library construction was performed using a SureSelect XT HS2 mRNA Library Preparation Kit (Agilent, Santa Clara, CA, USA) with up to 1 µg of total RNA input, according to the manufacturer’s protocol. The raw Fastq files and the processed filtered count matrix for mRNA sequencing were deposited at the NCBI GEO database under the accession number GSE196900. Partek Flow software (Build version 10.0.22.0121; Partek Inc., St. Louis, MO, USA) was used for analyzing the bulk RNA-sequencing data generated by Illumina NovaSeq 6000. First, base calling was performed with Illumina Real-Time Analysis software (RTA3, v3.4.4) and the FASTQ files were generated with bcl2fastq (version 2.20.0.422). Fastq files were trimmed from both ends with Phred quality scores lower than 35 before alignment. The quality scores of sequence reads were mapped to the human genome GRCh38 by STAR 2.7.8a with default parameters [34]. After alignment, the final BAM files were quantified using the Partek E/M algorithm [35] and annotated using Ensembl Transcripts release 102 to generate gene counts. The gene counts were normalized and further differential gene expression analysis was performed using DESeq2 between 5FUR and parental CRC cells [36]. Differentially expressed genes were selected based on log_2_ fold changes >1 and <−1 and a *p*-value of <0.05. For the identified DEGs, we performed pathway analysis using Qiagen IPA to identify gene ontologies, pathways, and the regulatory networks to which DE genes belong to, as well as upstream regulators. The canonical pathway analyses will further depict the DE genes by activation/inhibition through the calculation of a Z score, i.e., a statistical measure of the match between expected relationship direction between the regulator and its targets and observed gene expression comparing 5FUR and parental CRC cells.

### 2.15. Statistical Analysis

Statistical analyses were performed for three independent experiments using GraphPad Prism 8 software. All data are represented as the mean ± standard deviation of three independent experiments. A Student’s *t*-test was performed to assess statistical significance between two groups. A *p*-value of <0.05 was considered statistically significant.

## 3. Results

### 3.1. Metformin Treatment Inhibits Cell Proliferation and Colony Formation in CRC Cells

**Figure 1:**
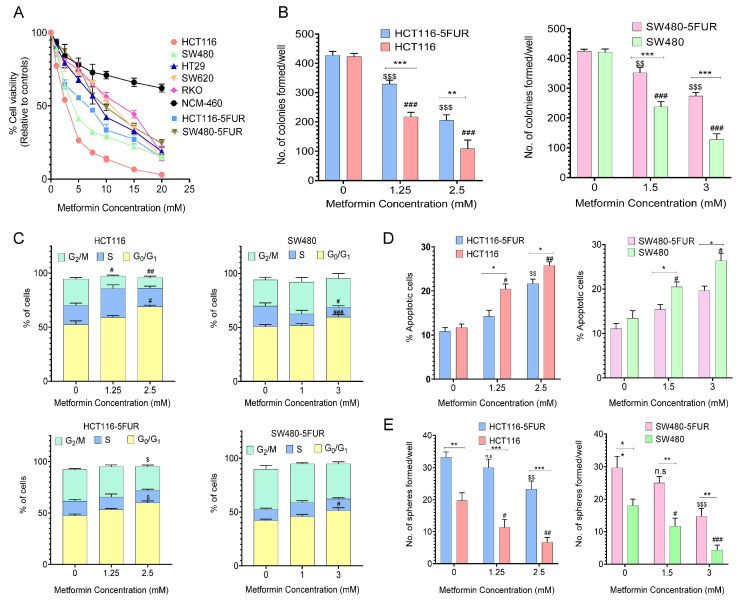
Anti-proliferative effect of metformin in parental and 5FUR CRC cells. (**A**) Measurement of percentage cell viability after treatment with metformin (1–20 mM) for 48 h in a panel of CRC cell lines by CCK-8 assay. (**B**) Colony-forming ability of parental and 5FUR CRC cells after treatment with different concentrations of metformin for 48 h. (**C**) Cell cycle analysis and (**D**) apoptosis measurement in parental and 5FUR CRC cells after treatment with different concentrations of metformin for 48 h. (**E**) Spheroid-formation capacity of parental and 5FUR CRC cells after treatment with metformin for 48 h. Statistical significance was determined by Student’s *t*-test. (Comparison between Parental vs. 5FU-resistant-* *p* < 0.05, ** *p* < 0.01, *** *p* < 0.001; comparison between Control and treatment groups for HCT116 and SW480-^$^ *p* < 0.05, ^$$^ *p* < 0.01, ^$$$^ *p* < 0.001; comparison between Control and treatment groups for HCT116-5FUR and SW480-5FUR-^#^ *p* < 0.05, ^##^ *p* < 0.01, ^###^ *p* < 0.001.), n.s. not significant.

In order to assess the effect of metformin treatment on cell proliferation in CRC cells we performed cell viability assays using a CCK-8 kit, following metformin treatment in a panel of CRC cells along with two 5FUR paired counterparts. We observed that 48 h of metformin treatment decreased cell viability in a dose-dependent manner in CRC cells, such as HCT116, HCT116-5FU-resistant (5FUR), SW480, SW480-5FUR, HT29, RKO, and SW620 (Figure 1A). The IC_50_ of metformin in a panel of CRC cell lines is presented in Supplementary Table S3. As expected, the IC_50_ values for metformin in 5FUR cells was much higher than their parental counterparts. More specifically, the IC_50_ value for metformin in HCT116 was 2.5 mM, while that of HCT116-5FUR was 5 mM; likewise, the IC_50_ for metformin in SW480 was 3 mM, while that of SW480 5FUR was 7.6 mM. Interestingly, we observed that, compared to all CRC cell lines, metformin treatment was safe; even at the highest concentration of 20 mM, the normal colonic epithelial cell line NCM460 did not exhibit a 50% decrease in cell viability (Figure 1A). We also observed that metformin treatment significantly decreased the colony-forming ability in both parental as well as 5FUR CRC cells (*p*-value < 0.01; Figure 1B and Supplementary Figure S1A). These data collectively demonstrate that metformin inhibits cell proliferation in CRC cells, which also corroborated previous findings [37,38,39], and this inhibition is more pronounced in parental CRC cells as compared to their 5FU-resistant counterparts.

### 3.2. Metformin Treatment Induces Cell Cycle Arrest and Apoptosis and Inhibits Spheroid Formation in CRC Cells

Next, we interrogated the cell cycle dynamics and apoptosis in both parental and 5FUR CRC cells, following treatment with metformin. It was observed that 2.5 mM of metformin treatment induced cell cycle arrest at the G_0_/G_1_ phase in parental as well as HCT116-5FUR cells (parental-untreated 52.55% vs. 2.5mM metformin 69.15%, *p* < 0.05; 5FUR-untreated 47.4% vs. 2.5mM metformin 58.05%, *p* < 0.05; Figure 1C). Similar results were observed for SW480 parental and 5FUR cells. 

Furthermore, we observed that metformin treatment significantly altered the percentage of apoptotic cells when compared to their untreated counterparts in parental as well as 5FUR CRC cells. For instance, 2.5 mM of metformin treatment exerted a significant increase in the fraction of apoptotic cells in parental and HCT116-5FUR cells (untreated 11.70% vs. 2.5 mM 25.725%, *p* < 0.01; 5FUR-untreated 10.85% vs. 2.5 mM 21.65%, *p* < 0.01; Figure 1D). We observed that metformin treatment significantly decreased the number of spheroids formed in parental CRC cells with increasing metformin concentrations (*p* < 0.01–0.05), whereas higher concentrations of metformin (2.5 mM) significantly decreased sphere formation in 5FUR CRC cells as compared to the parental cells (*p* < 0.01; Figure 1E and Supplementary Figure S1B). Collectively, these results suggest that metformin exhibits an anti-proliferative effect in CRC cells through cell cycle arrest, induction of apoptosis, and reduced spheroid-forming ability.

### 3.3. Transcriptomic Analysis Revealed Enrichment of Wnt/β-Catenin Signaling in 5FU-Resistant CRC Cells

**Figure 2:**
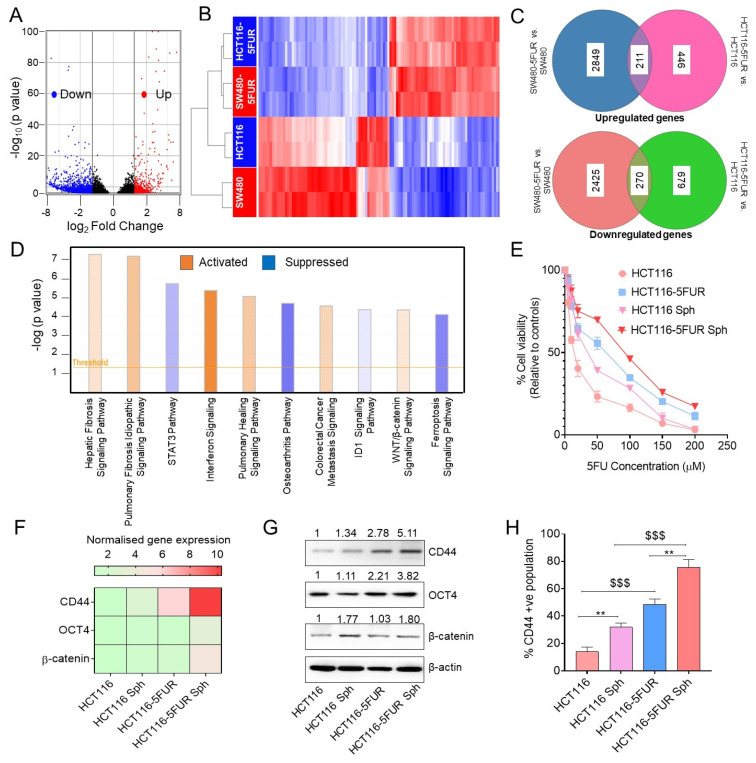
Genome-wide transcriptomic analysis of parental and 5FUR CRC cells and the role of CSCs in promoting chemoresistance in CRC. (**A**) Volcano plot showing differentially expressed genes between parental and 5FUR CRC cells based on log_2_FC > 1 and <−1 and a *p*-value of <0.05. (Red-upregulated, blue-downregulated, black-non-significant genes.) (**B**) Heatmap of differentially expressed genes in parental and 5FUR CRC cells. (**C**) Venn diagram analysis represents significant up- and downregulated genes in SW480-5FUR vs. SW480 and HCT116-5FUR vs. HCT116 compared to cells. (**D**) Ingenuity pathway analysis to identify the top 10 significantly activated and suppressed pathways in 5FUR and parental cells based on their −log *p*-value and Z score. (Orange-positive Z score, blue-negative Z score.) (**E**) Comparison of percentage cell viability of parental and 5FUR CRC cells and their corresponding spheroids after treatment with different concentrations of 5FU (5–200 µM). (**F**) Gene and (**G**) protein expression analysis of CSC markers (CD44, OCT4) and β-catenin in parental and HCT116-5FUR cells and their respective spheroid counterparts. Original blots see Figure S2. (**H**) Flow cytometric analysis of the CD44-positive population in parental and HCT116-5FUR cells and their corresponding 3D spheroids. Statistical significance was determined by a Student’s *t*-test. (Comparison between HCT116 vs. HCT116 spheroids and HCT116-5FUR vs. HCT116-5FUR spheroids-** *p* < 0.01; comparison between HCT116 vs. HCT116-5FUR and HCT116 spheroids and HCT116-5FUR spheroids-^$$$^ *p* < 0.001; P = parental CRC cells, R = 5FU-resistant CRC cells.)

Next, to decipher the molecular pathways associated with chemoresistance in CRC cells, we performed a genome-wide transcriptomic analysis using RNA-seq in parental and 5FU-resistant HCT116 and SW480 cell lines. The volcano plot illustrated upregulated (red) and downregulated genes (blue) based on their log_2_FC > 1 and <−1 and *p*-value of <0.05, represented as a heatmap in Figure 2B. In addition, the top 10 upregulated and downregulated genes between 5FUR and parental cells are presented in Supplementary Table S4 and the final list of significantly and differentially expressed genes in 5FU-resistant and parental CRC cells are presented in Supplementary Table S5. Furthermore, Venn diagrams represent the number of genes up- and downregulated in HCT116-5FUR vs. HCT116 compared with SW480-5FUR vs. SW480 (Figure 2C). It was observed that 211 upregulated and 270 downregulated genes overlapped between HCT116 and SW480 5FUR cells as compared to the parental cells. 

Furthermore, we performed an ingenuity pathway analysis (IPA) based on the differentially expressed genes between 5FUR and parental CRC cells. We selected the top 10 significant signaling pathways based on their −log (*p*-value). The activation or suppression status of the signaling pathways were determined based on their Z score. The positive Z score (orange) highlights the activated pathways and the negative Z score (blue) represents suppressed pathways between 5FUR and parental cells. It was observed that several important cancer-associated signaling pathways, including the Wnt signaling pathway, colorectal cancer metastasis signaling, etc., were activated, and the STAT3 pathway, ferroptosis, etc., were significantly suppressed between 5FUR CRC and parental cells (Figure 2D). In our study, we observed that Wnt/β-catenin signaling pathway is enriched and activated in 5FUR cells, and this pathway has been implicated in the maintenance of the CSC phenotype as promoting chemoresistance [21]. Therefore, we generated three-dimensional (3D) spheroid models, which are considered to possess an enriched population of stem-like cells [40,41], to evaluate the role of CSCs in promoting chemoresistance. We examined the chemoresistance attributes of the 3D spheroids using a CCK-8 assay after treatment with different concentrations of 5-flurouracil (5-FU). We observed that the spheroidal population of parental and HCT116-5FUR cells exhibited increased chemoresistance towards 5FU (Figure 2E). Furthermore, we evaluated the expression of stem-like cell markers, such as CD44 and OCT4, along with β-catenin, which is considered to be one of the critical components of the Wnt signaling pathway [32]. We observed that 5FU-resistant cells and their respective spheroid counterparts exhibited increased gene and protein expression of CD44, OCT4, and β-catenin (Figure 2F,G), along with an increased percentage of CD44-positive populations as compared to parental CRC cells (Figure 2H). These results also highlighted the possible role of cell stemness in promoting chemoresistance in CRC.

### 3.4. Selective Targeting of the Wnt Signaling Pathway by ICG-001 Promotes Chemosensitivity in CRC Cells

**Figure 3:**
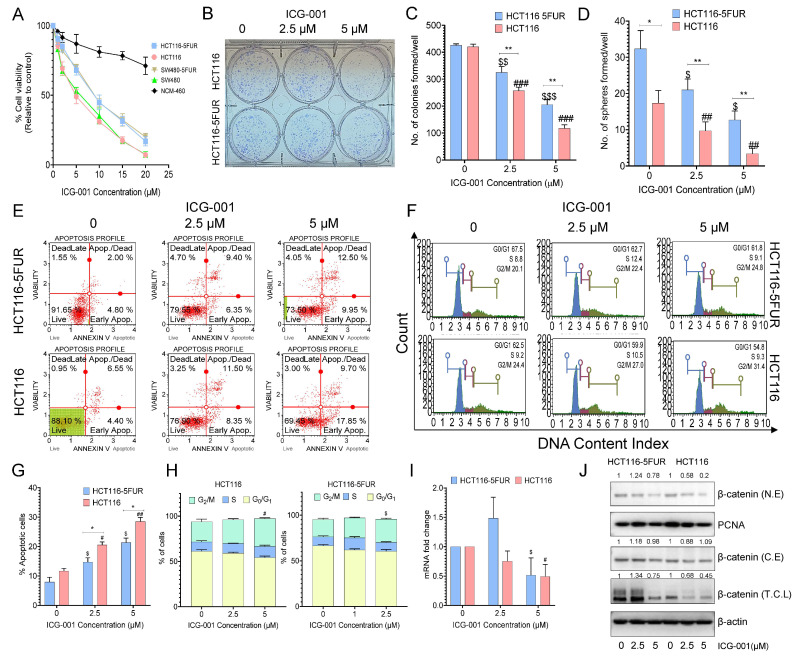
Anti-proliferative effects of the Wnt signaling inhibitor ICG-001 in parental and 5FUR CRC cells. (**A**) Measurement of percent cell viability after treatment with ICG-001 (1–20 mM) for 48 h in parental and 5FUR CRC cells by CCK-8 assay. (**B**) Representative images of colonies and (**C**) the number of colonies formed after treatment with different concentrations of ICG-001 for 48 h. (**D**) Sphere-formation capacity after treatment with ICG-001 for 48 h. (**E**). Representative histogram of the percentage of apoptotic cells and (**G**) a graphical representation of the percentage of apoptotic cells after treatment with different concentrations of ICG-001 in parental and HCT116-5FUR cells for 48 h. (**F**) Representative histograms of different phases of the cell cycle and (**H**) a graphical representation of the percentage of cells in different phases of the cell cycle after treatment with different concentrations of ICG-001 for 48 h. (**I**) Gene and (**J**) protein expression analyses of β-catenin in parental and HCT116-5FUR cells after treatment with ICG-001 for 48 h (N.E-nuclear extract, C.E-cytoplasmic extract, T.C.L-total cell lysates). Original blots see Figure S2. Statistical significance was determined by a Student’s *t*-test. (Comparison between Parental vs. 5FU-resistant group-* *p* < 0.05, ** *p* < 0.01; comparison between Control and treatment groups for HCT116-^$^ *p* < 0.05, ^$$^ *p* < 0.01, ^$$$^ *p* < 0.001; comparison between Control and treatment groups for HCT116-5FUR-^#^ *p* < 0.05, ^##^ *p* < 0.01, ^###^ *p* < 0.001.)

Previous studies have highlighted that the Wnt signaling pathway is implicated in promoting the CSC phenotype [21,32], which was once again confirmed in our transcriptomic analysis, where we also observed that this signaling pathway is enriched in 5FU-resistant CRC cells. Therefore, we performed selective targeting of the Wnt signaling pathway in CRC cells by using ICG-001, a small molecule inhibitor that targets the Wnt signaling pathway by inhibiting TCF/β-catenin-mediated transcription and evaluated its anti-proliferative role in parental and 5FUR CRC cell lines. Interestingly, we observed that ICG-001 effectively decreased cell viability in parental and resistant cells in a dose-dependent manner, with IC_50_ values of 4.5 and 4.7 μM for parental HCT116 and SW480 cells and 6.5 and 5.6 μM for their respective 5FUR counterparts (Figure 3A). A significant reduction in the colony-forming and sphere-forming capacities of CRC cells was observed following treatment with ICG-001 (*p* < 0.01–0.001; 2.5 and 5 μM; Figure 3B–D). We also observed that increasing concentrations of ICG-001 treatment promoted apoptosis significantly (*p* < 0.05; Figure 3E,G). Cell cycle analysis of parental and resistant cells revealed that resistant and parental CRC cells exhibited an increased G_2_/M phase arrest after ICG-001 treatment (Figure 3F,H). Furthermore, we also confirmed that ICG-001 treatment decreased the gene expression of β-catenin (Figure 3I). Next, we extracted the nuclear and cytoplasmic protein fraction to check the nuclear localization of β-catenin after treatment with ICG-001. We observed that ICG-001 treatment significantly decreased the nuclear localization of β-catenin, as is evident from the decreased expression of β-catenin in nuclear extracts. The effect was more pronounced in HCT116 parental cells as compared to 5FUR counterparts (Figure 3J). Taken together, these results highlighted the anti-proliferative effects of ICG-001 by decreasing the expression of β-catenin in parental as well as 5FUR CRC cells. 

### 3.5. Combined Treatment with Metformin and ICG-001 Abrogates Chemoresistance in a Synergistic Manner

**Figure 4:**
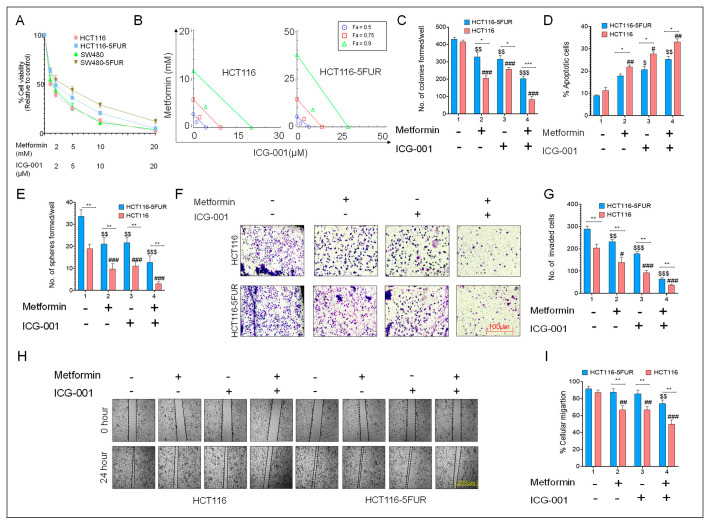
Combined effect of metformin and ICG-001 in parental and 5FUR CRC cells. (**A**) Measurement of percentage cell viability using a CCK-8 assay after treatment with metformin (1–20 mM) and ICG-001 in parental and HCT116-5FUR cells for 48 h. (**B**) Isobologram analysis to determine the mechanism of action of metformin and ICG-01 in parental and HCT116-5FUR cells. (**C**) Colony-forming ability and (**D**) measurement of the percentage of apoptotic cells. (**E**) Sphere-forming ability after treatment with metformin and ICG-001 for 48 h, either alone or in combination, in parental and 5FUR CRC cells. (**F**) Representative images and (**G**) a graphical representation of the number of invaded cells after treatment with metformin and ICG-001 for 48 h, either alone or in combination. (**H**) Representative images of wound and (**I**) a graphical representation of percentage of wound closure after 24 h of treatment with metformin and ICG-001, either alone or in combination. Statistical significance was determined by a Student’s *t*-test. (Comparison between Parental vs. 5FUR-* *p* < 0.05, ** *p* < 0.01, *** *p* < 0.001; comparison between Control and treatment groups for HCT116-^$^ *p* < 0.05, ^$$^ *p* < 0.01, ^$$$^ *p* < 0.001; comparison between Control and treatment groups for HCT116-5FUR-^#^ *p* < 0.05, ^##^ *p* < 0.01, ^###^ *p* < 0.001.).

Next, in order to determine whether metformin has any synergistic activity together with ICG-001 in promoting chemosensitivity, we evaluated the combinatorial effect of metformin and ICG-001 treatment in parental and 5FU-resistant HCT116 cells. We observed that combined treatment with these two drugs decreased cell viability at much lower IC_50_ concentrations compared to the treatment with individual drugs (Figure 4A). Next, we performed an isobologram analysis to evaluate the mechanism of action of these two drugs and observed that metformin and ICG-001 act synergistically to decrease cell viability in parental and 5FU-resistant CRC cells as is evident from their combination index (CI) of <1 (Figure 4B). The combination treatment also exhibited an increased percentage of apoptotic cells and decreased the number of spheroids formed in parental and 5FU-resistant CRC cells (Figure 4C,D). Furthermore, we observed that the combined treatment decreased the invasion capacity of parental and 5FU-resistant cells vs. individual treatments with either metformin or ICG-001 (Figure 4F,G). A wound healing assay also showed a decreased percentage of cellular migration in CRC cells after combined treatment of metformin and ICG-001 (Figure 4H,I), highlighting that metformin and ICG-001 chemosensitize CRC cells in a synergistic manner.

### 3.6. Combined Treatment with Metformin and ICG-001 Decreases the Expression of CSC Markers and Promotes Autophagy and Apoptosis in CRC Cells 

**Figure 5:**
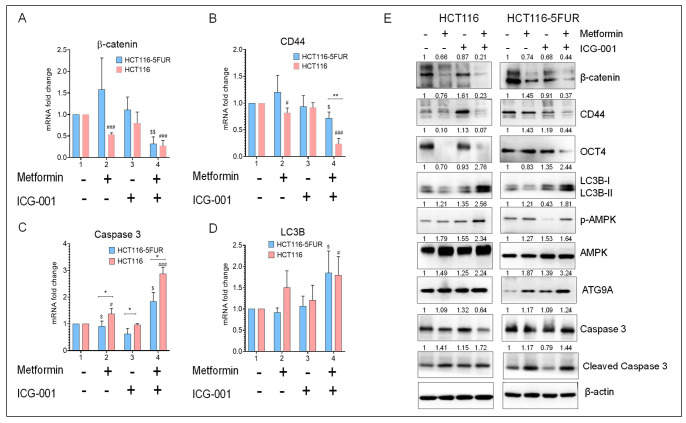
Gene and protein expression profiling after treatment with metformin and ICG-001, either alone or in combination, in parental and 5FUR CRC cells. Gene expression of (**A**) β-catenin, (**B**) CD44, (**C**) caspase 3 and (**D**) LC3B after treatment with metformin and ICG-001, either alone or in combination, for 48 h. β-actin was used as a housekeeping gene. (**E**) WB analysis of β-catenin, CSC markers (CD44 and OCT4), apoptosis markers (caspase 3 and cleaved caspase 3), autophagy markers (LC3B, ATG9A), and phosphorylation status of AMPK after treatment with metformin and ICG-001, either alone or in combination, for 48 h. β-actin was used as loading control for WB analysis. Original blots see Supplementary File. Statistical significance was determined by a Student’s *t*-test. (Comparison between Parental vs. 5FUR-* *p* < 0.05, ** *p* < 0.01; Comparison between Control and treatment groups for HCT116-^$^ *p* < 0.05, ^$$^ *p* < 0.01; Comparison between Control and treatment groups for HCT116-5FUR-^#^ *p* < 0.05, ^###^ *p* < 0.001.).

Next, we explored the effect of the combined treatment with metformin and ICG-001 on the gene expression of markers associated with regulating the pluripotency of stem cells, such as β-catenin, CD44, an apoptotic marker (caspase 3), and an autophagy marker (LC3B), in parental and 5FU-resistant HCT116 cells. It was observed that the combination treatment resulted in increased expression of caspase 3 and LC3B and decreased expression of the CSC marker (CD44) and β-catenin (*p* < 0.01–0.05; Figure 5A–D). Consistent with the gene expression data, Western blot analysis also showed increased expression of autophagy markers, such as LC3B and ATG9A, along with increased phosphorylation of AMPK. It was also observed that the combined treatment of metformin and ICG-001 decreased the expression of CSC markers, such as CD44 and OCT4, along with β-catenin. We also observed increased expression of apoptotic markers, such as cleaved caspase 3, after combinatorial treatment (Figure 5E). Collectively, these results indicate that the combined treatment with metformin and ICG-001 promotes autophagy and apoptosis in CRC cells by abrogating the stemness phenotype by suppressing Wnt/β-catenin signaling.

### 3.7. Metformin and ICG-001 Promote Anti-Tumor Activity in CRC Patient-Derived Organoid Models

**Figure 6:**
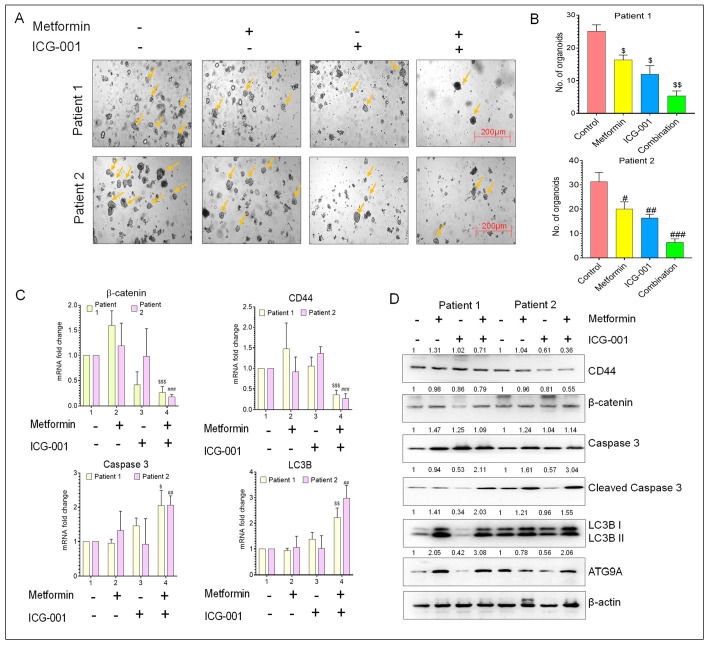
The anti-tumor activity of metformin and ICG-001 in a CRC patient-derived organoid model. (**A**) Representative images of patient-derived tumor organoids. (**B**) Number of organoids formed after treatment with metformin and ICG-001, either alone or in combination. (**C**) Gene expression analysis of β-catenin, CSC marker (CD44), apoptosis marker (caspase 3) and autophagy marker (LC3B) in patient-derived tumor organoid after treatment with metformin and ICG-001, either alone or in combination, for 10 days. β-actin was used as a housekeeping gene. (**D**) WB analysis of β-catenin, CSC marker (CD44), apoptosis markers (caspase 3 and cleaved caspase 3), and autophagy markers (LC3B, ATG9A) from the protein lysate extracted from patient-derived organoids after treatment with metformin and ICG-001, either alone or in combination. β-actin was used as loading control for WB analysis. Original blots see Supplementary File. Statistical significance was determined by a Student’s *t*-test. (Comparison between Control vs. treatment groups for Patient 1-^$^ *p* < 0.05, ^$$^ *p* < 0.01, and ^$$$^ *p* < 0.001; comparison between Control vs. treatment groups for Patient 2-^#^ *p* < 0.05, ^##^ *p* < 0.01, and ^###^ *p* < 0.001.).

To further confirm the above observations from cell culture experiments, we evaluated the anti-tumor activity of metformin and ICG-001, individually or in combination, in tumor-derived organoid models generated from patients with advanced CRC. The tumor organoids were cultured in a medium containing appropriate concentrations of metformin and ICG-001, either alone or in combination, for 10 days. In accordance with the results obtained from cell lines, the combined treatment with metformin and ICG-001 significantly decreased the number of patient-derived tumor organoids vs. when treated with individual drugs (Figure 6A,B). Furthermore, we observed that combined treatment significantly decreased the gene expression of the CSC marker CD44 along with β-catenin and increased the expression of the autophagy marker LC3B and apoptosis marker caspase 3 (Figure 6C). Western blot analysis also revealed an increased expression of autophagy markers (LC3B and ATG9A), apoptosis marker (cleaved caspase 3), along with the decreased expression of the CSC marker CD44 and β-catenin (Figure 6D). The above data indicate that combined treatment with metformin and ICG-001 exert anti-tumor activity in a patient-derived organoid model by promoting autophagy and apoptosis by decreasing the activation of Wnt/β-catenin signaling, which subsequently reduces the CSC phenotype.

## 4. Discussion

Considerable advancements and improvements in treatment modalities for CRC has led to increased overall survival among CRC patients in the past decade [42]. Since 1990, 5FU-based chemotherapy has been the backbone of the CRC treatment regimen [43]. A thymidylate synthase inhibitor, 5FU exerts its anti-tumor efficacy by increasing DNA damage, thereby causing cell cycle arrest and apoptosis [44,45]. Despite its routine use, the beneficial role of 5FU is somewhat limited as its treatment is often associated with considerable side effects, such nausea, vomiting, diarrhea, increased risk of infection, anemia, etc. [46]. Over the past few decades, there has been a growing interest in exploring the use of various phytochemicals from natural products as potential anti-cancer agents due to their reduced systemic toxicity [26,27,47]. Several studies have shown that dietary phytochemicals exert anti-tumor efficacy by reducing the proliferation of cancer cells by promoting cell cycle arrest and apoptosis [26,28,48]. Metformin, a hypoglycemic agent derived from a natural compound found in French lilac has been reported to exert anti-tumor effects in different solid tumors [11]. 

In this study, we first evaluated the effect of metformin treatment in a panel of CRC cell lines along with their 5FU-resistant counterparts. We observed that metformin treatment exhibited an anti-proliferative effect in CRC cells by promoting apoptosis and cell cycle arrest at the G_0/_G_1_ phase and that this effect was more pronounced in parental CRC cells as compared to 5FUR CRC cells. These findings led us to perform genome-wide transcriptomic profiling to identify differentially expressed genes and associated pathways between these two cell types. The transcriptomic analysis revealed significantly upregulated genes in 5FUR and parental CRC cells that were associated with pathways associated with Wnt/β-catenin signaling, colorectal cancer metastases, etc. Previous reports also highlighted the role of the Wnt signaling pathway in the maintenance of the CSC phenotype and its role in promoting 5FU resistance in CRC due to aberrant activation [49,50]. It has been reported that one of the primary factors underpinning the development of therapeutic resistance is the presence of CSCs in the bulk of tumors [20,21]. Previous studies have also demonstrated that certain cancer cells could re-acquire cancer stem cell (CSC) traits via intrinsic stem-associated gene expression and the extrinsic tumor microenvironment [20,51,52,53]. Therefore, in this study, to elucidate the role of CSCs in promoting therapeutic resistance, we developed a spheroid model which represents a CSC population [20,53]. Over the past few decades, many 3D culture methods of CSCs in the form of spheres have been developed [53]. In this study we have employed the most commonly used method for forming 3D spheroids by using scaffold-free methods, such as the ultra-low attachment plate method, using serum free conditions along with growth factors. A subgroup of tumor cells which can survive in a serum-free culture was identified and isolated from a group of tumor cells, which was further used to form a tumorsphere. Previous studies demonstrated that these cells could proliferate and expand clonally in the absence of serum supplements, suggesting that they may have stem cell-like features [20,21,52]. In accordance with a previous study carried out by our group [20,27], in this study we also generated 3D spheroids and observed that the spheroids exhibit increased expression of CSC markers, such as CD44, OCT4, and β-catenin, along with an increased percentage of CD44-positive populations. We observed that 3D spheroids were resistant to 5FU, which corroborated our previous findings [20]. We also observed that HCT116-5FUR and SW480 cells exhibited increased sphere-forming abilities as compared with their parental counterparts. This highlighted the possible role of CSCs in promoting therapeutic resistance in CRC and the urgent need to develop novel therapeutic strategies which can reduce the toxicity associated with chemotherapeutic drugs and target CSC populations to prevent disease relapse in CRC. Therefore, in this study we further investigated the combined effect of metformin and ICG-001, which has been reported as a Wnt signaling inhibitor [54], in abrogating CSC-mediated chemoresistance in CRC.

ICG-001 has been reported as an antagonist for Wnt/β-catenin/TCF-mediated transcription by which means it blocks the activation of downstream targets required for cell proliferation and tumor development by promoting apoptosis, reducing chemoresistance mediated by CSCs [54,55]. TCF74 is an important regulator of the canonical Wnt/β-catenin signaling pathway and was reported to be frequently overexpressed in rectal cancers and resistant to preoperative 5FU-based long-term chemoradiotherapy [56,57]. Therefore, it is necessary to generate safe and effective molecules targeting the Wnt/β-catenin-signaling pathway in CRC. In this study we observed that ICG-001 treatment exhibited anti-proliferative effects by promoting apoptosis in parental and 5FUR CRC cells. Consistent with the anti-proliferative effect of metformin, ICG-001 also exhibited a more pronounced cytotoxic effect in parental CRC cells as compared to 5FUR CRC cells. We also observed that ICG-001 blocks the nuclear translocation of β-catenin; the effect was significant in parental CRC cells as compared to 5FUR CRC cells after treatment with 2.5 µm of ICG-001. These findings led us to further investigate the combinatorial effect of metformin and ICG-001 treatment in parental and 5FU-resistant CRC cells along with CRC patient-derived organoid models. We observed that combined treatment with metformin and ICG-001 exerts an anti-proliferative effect in a synergistic manner and decreased the migration and invasion ability of parental and 5FUR CRC cells. Furthermore, we investigated the molecular mechanisms associated with the anti-proliferative effect of metformin and ICG-001 by investigating programmed cell death mechanisms, such as autophagy and apoptosis. 

Autophagy eliminates oncogenic protein substrates, toxic unfolded proteins, and damaged organelles in lysosomes [58,59]. Apoptosis is considered to be a defensive mechanism in the presence of toxic agents, generally characterized by distinct morphological properties, such as cellular shrinkage with nuclear chromatin condensation and nuclear fragmentation [60]. Previous studies showed that metformin triggers autophagy by AMPK activation, which leads to subsequent inhibition of mTOR signaling [61,62], as well as promoting apoptosis in cancer cells [63,64]. In addition, several studies also reported crosstalk between Wnt/β-catenin signaling and autophagy [65]. It was observed that inhibition of the Wnt/β-catenin pathway upregulates P62, LC3, and Beclin expression in different cancers, which subsequently increase autophagic flux [66,67]. In accordance with these previous studies, we also observed that combined treatment with metformin and ICG-001 increased the expression of autophagy markers, such as LC3B and ATG9A, and activated AMPK signaling pathway through increased phosphorylation of AMPK along with decreased expression of β-catenin. Furthermore, we also observed that the combined treatment increased the expression of apoptotic markers, such as cleaved caspase 3, and decreased the expression of CSC markers (CD44 and OCT4). 

The previous in vitro findings from this study were further confirmed using a patient-derived organoid model. The in vitro organoid model employs self-renewing tissues based on its stem cell characteristics [27,41]. Tumor organoids represent a tumor microenvironment which can resemble different cell lineages that reflect the structure and function of the organ whereas 3D spheroids represent heterogenous populations containing proliferating, quiescent, and necrotic cells, transiently resembling 3D cellular organization [68]. Therefore, tumor organoids are considered an important tool for studying tumor microenvironments, cellular interactions, and cellular responses after drug treatment, owing to their near physiological 3D architecture [68]. In this study, we observed that the combined treatment of metformin and ICG-001 exerts an anti-tumor effect, as was evident from the decreased number of organoids and the increased gene and protein expression of autophagy markers and decreased expression of CSC markers, along with β-catenin. Therefore, from this study we can speculate that metformin and ICG-001 activate autophagy and apoptosis by decreasing the activation of the Wnt/β-catenin signaling pathway. The anti-tumor effect of the combined treatment of metformin and ICG-001 highlighted its potential role as an alternative therapeutic strategy in CRC. 

We would like to acknowledge some of the limitations of the current study. First, it primarily consisted of just two (parental and 5FUR CRC) cell lines and none of them was RAS/BRAF wild type, which encompasses the majority of CRC specimens. Therefore, further study of RAS/BRAF wild type CRC cell lines is required. Second, we did not evaluate the effect of the combined treatment of metformin and ICG-001 in a mouse xenograft model. Third, in the patient-derived organoid model, we observed that the combined treatment exhibited a more significant decrease in the CSC phenotype in Patient 2 as compared to Patient 1. This might be due to the inherent tumoral heterogeneity between these two organoids. Lastly, as the purpose of this study was to evaluate the efficacy of the combined treatment of metformin and ICG-001 in abrogating CSC-mediated chemoresistance, we have not carried out a detailed mechanistic study to elucidate the possible mechanism of action of these two drugs. Further studies to characterize and dissect the molecular underpinnings of these two drugs in the abrogation of CSC-mediated chemoresistance are therefore warranted.

## 5. Conclusions

In conclusion, our findings suggest that a combined treatment with metformin and ICG-001 synergistically exerts cytotoxic potential by abrogating CSC-mediated chemoresistance in CRC. Moreover, the use of metformin along with CSC targeted therapy will reduce the adverse effects and the toxicity associated with conventional chemotherapy. Furthermore, since metformin is relatively inexpensive compared to other chemotherapy drugs, it may serve as a cost-effective and effective adjuvant treatment option along with conventional chemotherapy in patients suffering with colorectal cancer.

## Data Availability

The data presented in this study are available on request from the corresponding author.

## Supplementary Materials

The following supporting information can be downloaded at: https://www.mdpi.com/article/10.3390/cancers14051281/s1, Figure S1: (A) Colony forming assay and (B) spheroid forming assay after treatment with different concentrations of metformin for 48 h in HCT116 and SW480-5FUR and parental cells; Figure S2: Original blots; Table S1: Clinical details of the CRC patients used for tumor organoid generation; Table S2: List of primer sequences; Table S3: IC_50_ value of metformin and ICG-001 in different CRC cells; Table S4: List of top 10 upregulated and downregulated genes between 5FU-resistant and parental colorectal cancer cells. Table S5: List of significant differentially expressed genes between 5FUR and parental CRC cells obtained from DeSeq2 analysis.

## Abbreviations

CRC, colorectal cancer; CSCs, cancer stem-like cells; 5FU, 5-fluorouracil; T2DM, type 2 diabetes mellitus; PCOS, polycystic ovarian syndrome; OS, overall survival; DFS, disease-free survival; CI, combination index.
