# Correction: Roy et al. Metformin and ICG-001 Act Synergistically to Abrogate Cancer Stem Cells-Mediated Chemoresistance in Colorectal Cancer by Promoting Apoptosis and Autophagy. *Cancers* 2022, *14*, 1281

**DOI:** 10.3390/cancers17030528

**Published:** 2025-02-05

**Authors:** Souvick Roy, Yinghui Zhao, Yate-Ching Yuan, Ajay Goel

**Affiliations:** 1 Department of Molecular Diagnostics and Experimental Therapeutics, Beckman Research Institute of City of Hope, Monrovia, CA 91016, USA; soroy@coh.org (S.R.); yinghuizhao@mail.sdu.edu.cn (Y.Z.); 2 Department of Clinical Laboratory, The Second Hospital, Cheeloo College of Medicine, Shandong University, Jinan 250033, China; 3 Bioinformatics Core Facility, City of Hope National Medical Center, Duarte, CA 91010, USA; yyuan@coh.org; 4 Department of Medical Oncology, City of Hope National Medical Center, Duarte, CA 91010, USA; 5 City of Hope Comprehensive Cancer Center, Duarte, CA 91010, USA

In the original publication [1], there were errors in Figures 4F, 5E and 6A. In Figures 4F and 6A there were overlaps in representative images due to errors during the figure preparation process. In Figure 5E there was an error in sample annotation of Western blot images of cleaved caspase 3. The corrected Figure 4, Figure 5 and Figure 6 appear below. Additionally, the associated Supplementary Figure S2 has been updated. The authors state that the scientific conclusions are unaffected and apologize for any inconvenience caused. This correction was approved by the Academic Editor. The original publication has also been updated.

## Figures and Tables

**Figure 4:**
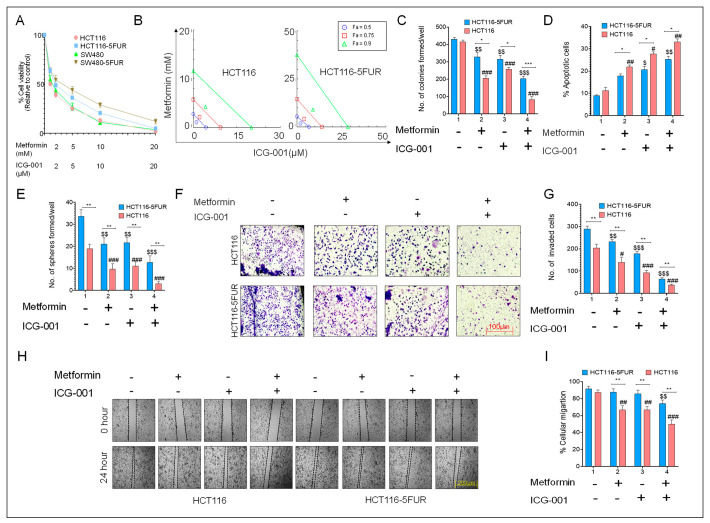
Combined effect of metformin and ICG-001 in parental and 5FUR CRC cells. (**A**) Measurement of percentage cell viability using a CCK-8 assay after treatment with metformin (1–20 mM) and ICG-001 in parental and HCT116-5FUR cells for 48 h. (**B**) Isobologram analysis to determine the mechanism of action of metformin and ICG-01 in parental and HCT116-5FUR cells. (**C**) Colony-forming ability and (**D**) measurement of the percentage of apoptotic cells. (**E**) Sphere-forming ability after treatment with metformin and ICG-001 for 48 h, either alone or in combination, in parental and 5FUR CRC cells. (**F**) Representative images and (**G**) a graphical representation of the number of invaded cells after treatment with metformin and ICG-001 for 48 h, either alone or in combination. (**H**) Representative images of wound and (**I**) a graphical representation of percentage of wound closure after 24 h of treatment with metformin and ICG-001, either alone or in combination. Statistical significance was determined by a Student’s *t*-test. (Comparison between Parental vs. 5FUR-* *p* < 0.05, ** *p* < 0.01, *** *p* < 0.001; comparison between Control and treatment groups for HCT116-^$^ *p* < 0.05, ^$$^ *p* < 0.01, ^$$$^ *p* < 0.001; comparison between Control and treatment groups for HCT116-5FUR-^#^ *p* < 0.05, ^##^ *p* < 0.01, ^###^ *p* < 0.001.).

**Figure 5:**
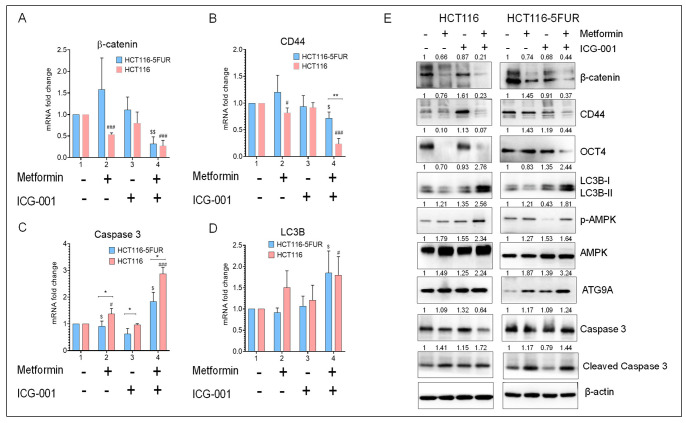
Gene and protein expression profiling after treatment with metformin and ICG-001, either alone or in combination, in parental and 5FUR CRC cells. Gene expression of (**A**) β-catenin, (**B**) CD44, (**C**) caspase 3 and (**D**) LC3B after treatment with metformin and ICG-001, either alone or in combination, for 48 h. β-actin was used as a housekeeping gene. (**E**) WB analysis of β-catenin, CSC markers (CD44 and OCT4), apoptosis markers (caspase 3 and cleaved caspase 3), autophagy markers (LC3B, ATG9A), and phosphorylation status of AMPK after treatment with metformin and ICG-001, either alone or in combination, for 48 h. β-actin was used as loading control for WB analysis. Original blots see Supplementary File. Statistical significance was determined by a Student’s *t*-test. (Comparison between Parental vs. 5FUR-* *p* < 0.05, ** *p* < 0.01; Comparison between Control and treatment groups for HCT116-^$^ *p* < 0.05, ^$$^ *p* < 0.01; Comparison between Control and treatment groups for HCT116-5FUR-^#^ *p* < 0.05, ^###^ *p* < 0.001.).

**Figure 6:**
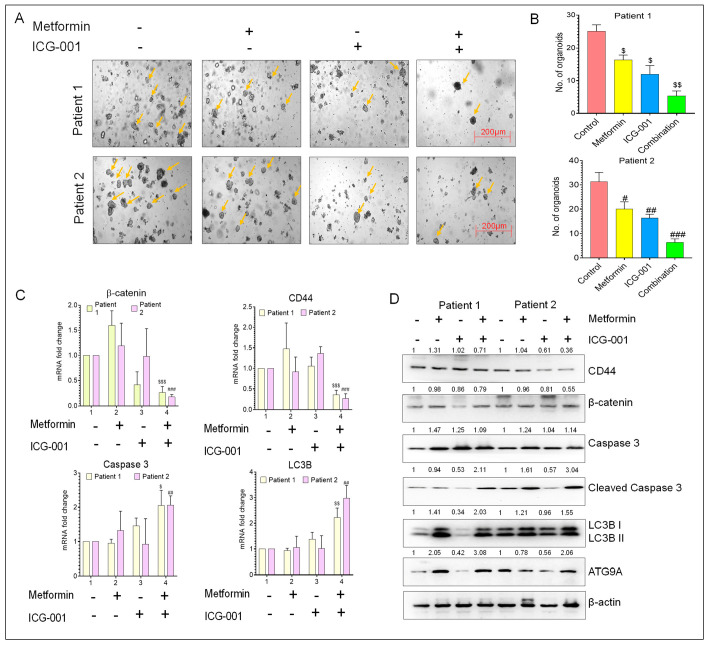
The anti-tumor activity of metformin and ICG-001 in a CRC patient-derived organoid model. (**A**) Representative images of patient-derived tumor organoids. (**B**) Number of organoids formed after treatment with metformin and ICG-001, either alone or in combination. (**C**) Gene expression analysis of β-catenin, CSC marker (CD44), apoptosis marker (caspase 3) and autophagy marker (LC3B) in patient-derived tumor organoid after treatment with metformin and ICG-001, either alone or in combination, for 10 days. β-actin was used as a housekeeping gene. (**D**) WB analysis of β-catenin, CSC marker (CD44), apoptosis markers (caspase 3 and cleaved caspase 3), and autophagy markers (LC3B, ATG9A) from the protein lysate extracted from patient-derived organoids after treatment with metformin and ICG-001, either alone or in combination. β-actin was used as loading control for WB analysis. Original blots see Supplementary File. Statistical significance was determined by a Student’s *t*-test. (Comparison between Control vs. treatment groups for Patient 1-^$^ *p* < 0.05, ^$$^ *p* < 0.01, and ^$$$^ *p* < 0.001; comparison between Control vs. treatment groups for Patient 2-^#^ *p* < 0.05, ^##^ *p* < 0.01, and ^###^ *p* < 0.001.).

**Figure S2:**
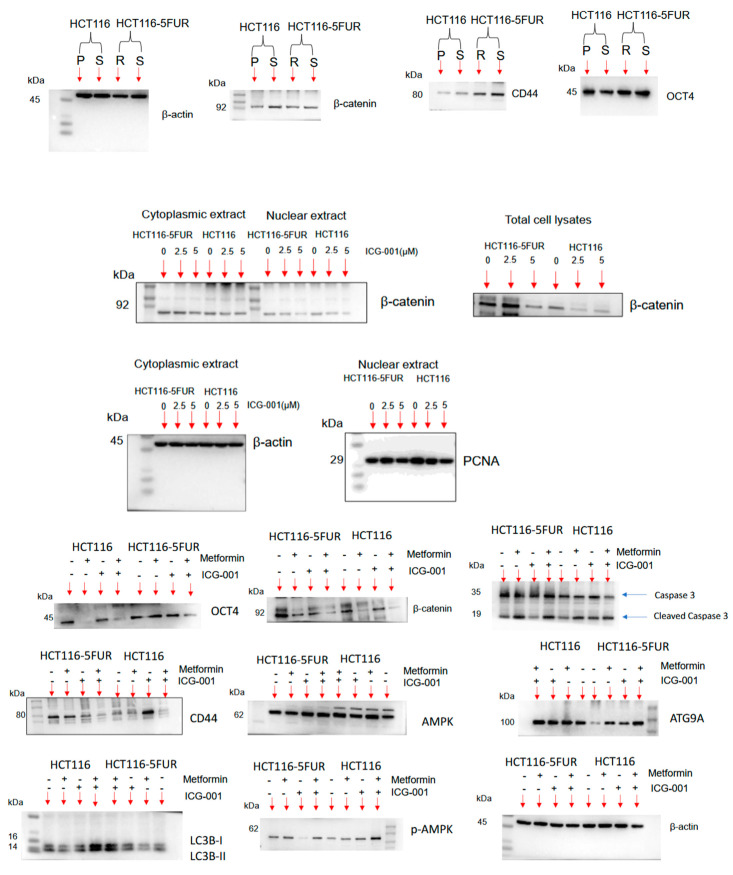

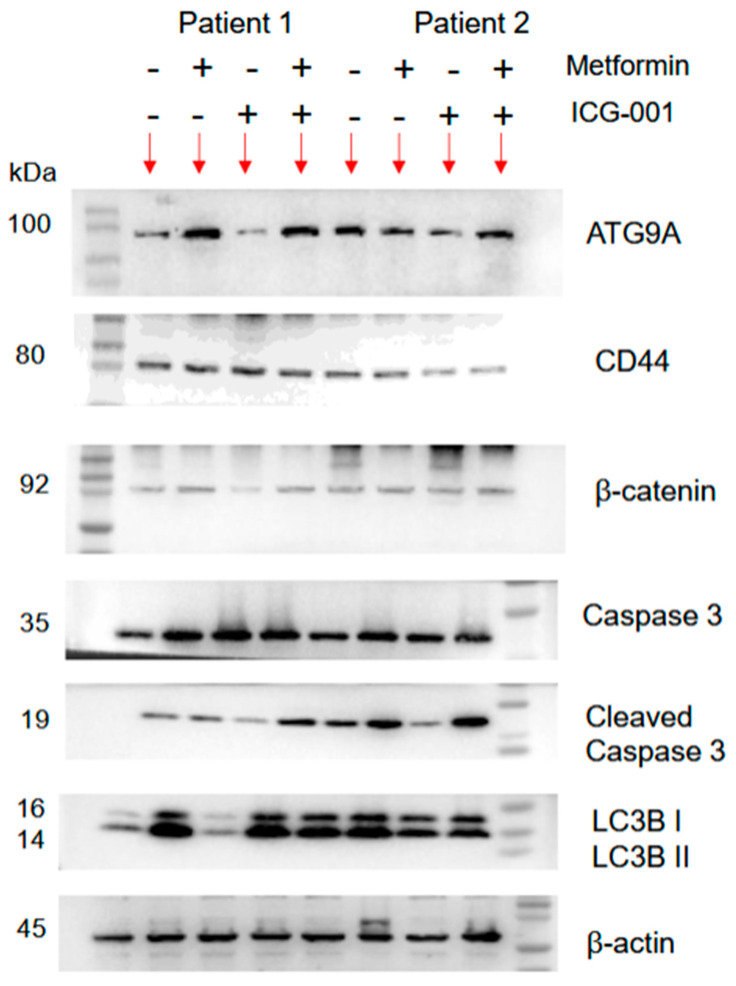
Original blots.
