# Supplementary material for: Metformin and ICG-001 Act Synergistically to Abrogate Cancer Stem Cells-Mediated Chemoresistance in Colorectal Cancer by Promoting Apoptosis and Autophagy
Source: Cancers (Basel). 2022 Mar 2;14(5):1281. doi: 10.3390/cancers14051281 (PMC8908991; doi:10.3390/cancers14051281)
Supplement: Supplementary file 1 [file cancers-14-01281-s001.zip › cancers-1529618-Supplementary.pdf]

Supplementary Material: Metformin and ICG-001 Act Synergistically to Abrogate Cancer Stem Cells-Mediated Chemoresistance in Colorectal Cancer by Promoting Apoptosis and Autophagy

Souvick Roy, Yinghui Zhao, Yate-Ching Yuan and Ajay Goel

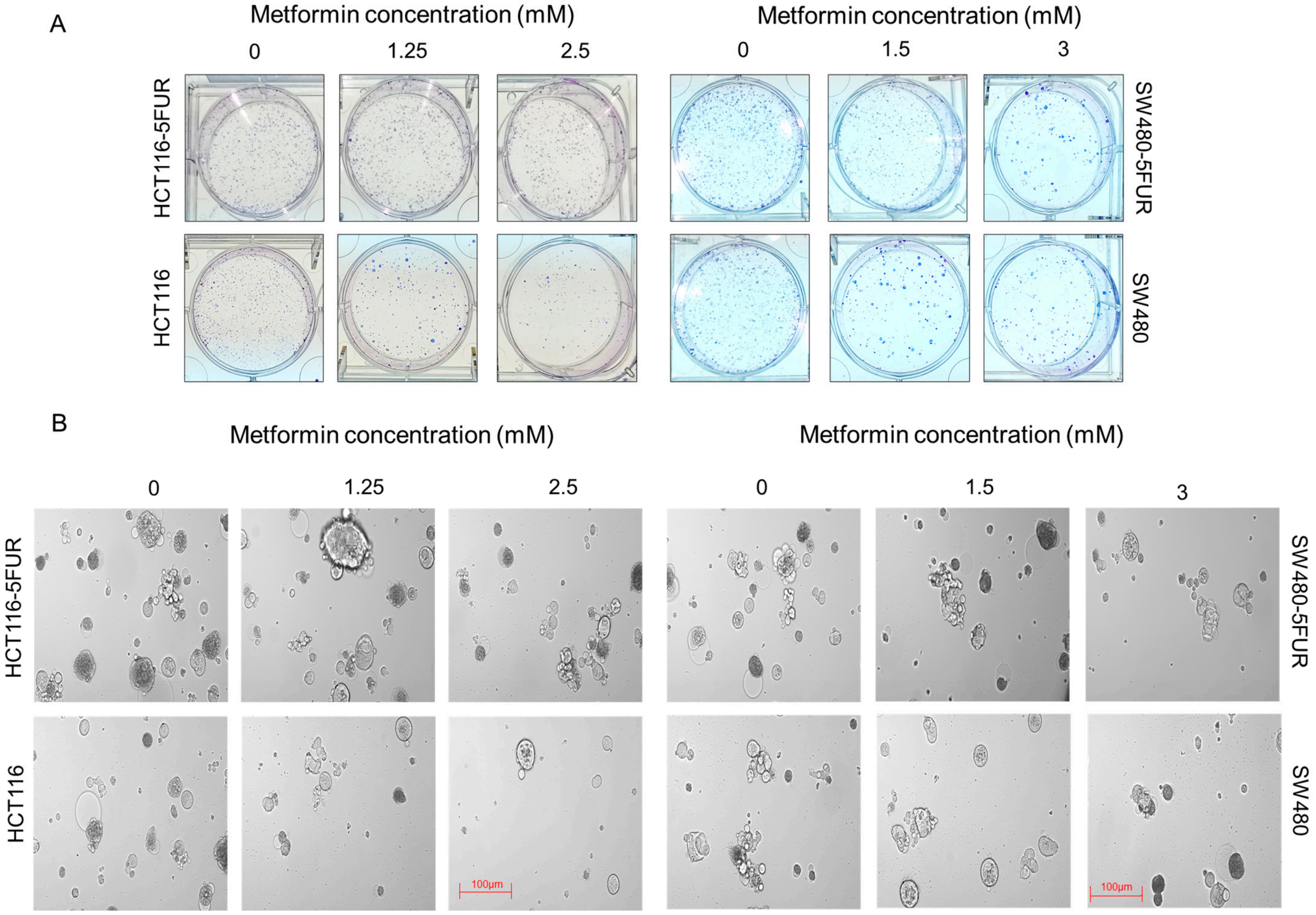

Figure S1. (A) Colony forming assay and (B) spheroid forming assay after treatment with different concentrations of metformin for 48 hours in HCT116 and SW480-5FUR and parental cells.

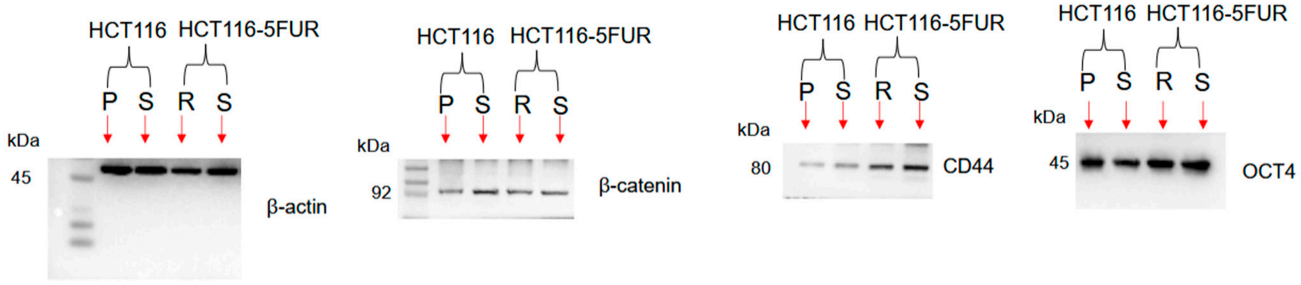

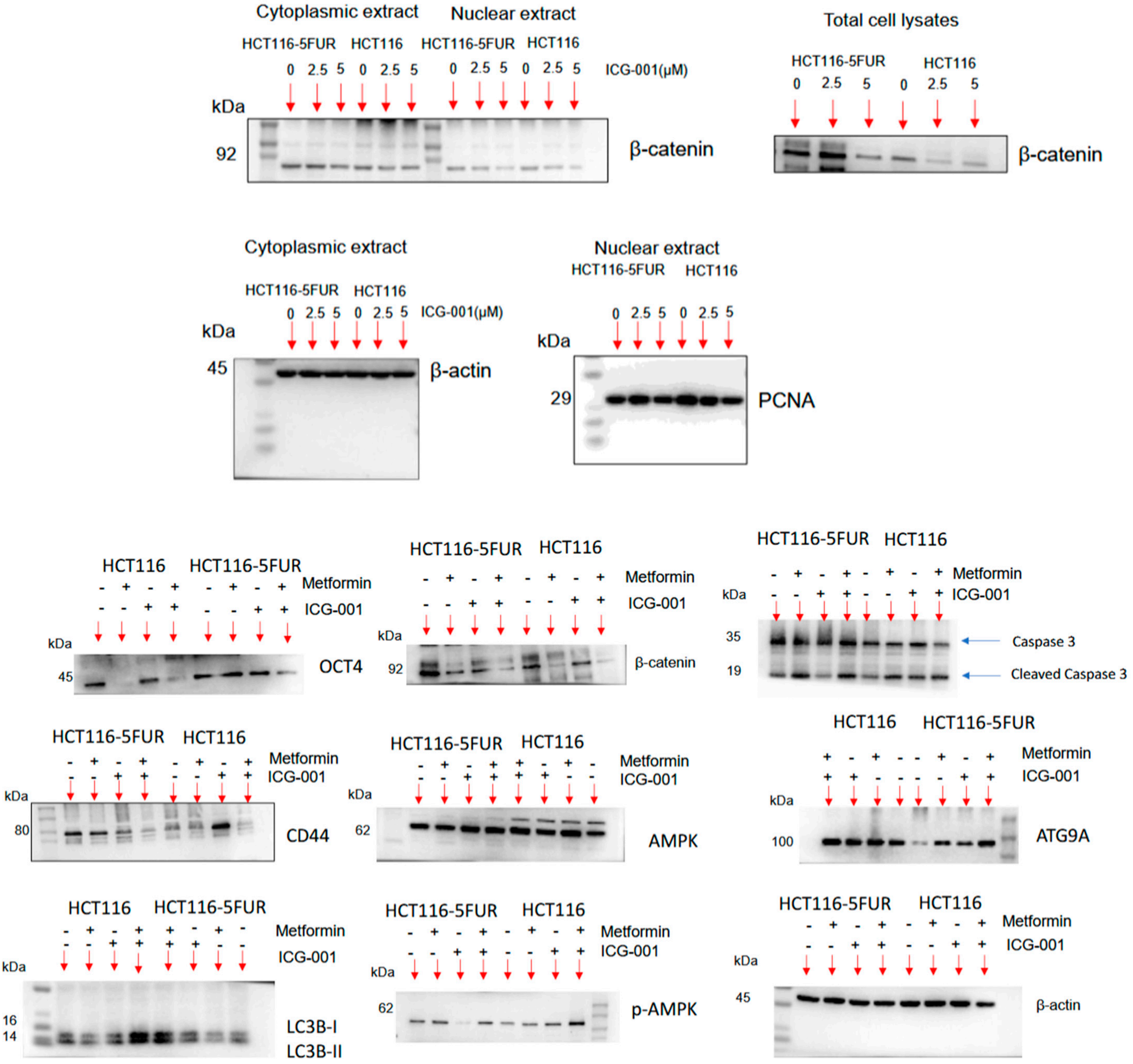
Cytoplasmic extract
Nuclear extract
HCT116-5FUR
HCT116
0 2.5 5 0 2.5 5 0 2.5 5 0 2.5 5
ICG-001(μM)
kDa
92
β-catenin
Total cell lysates
HCT116-5FUR
HCT116
0 2.5 5 0 2.5 5
β-catenin
Cytoplasmic extract
HCT116-5FUR
HCT116
0 2.5 5 0 2.5 5
ICG-001(μM)
kDa
45
β-actin
Nuclear extract
HCT116-5FUR
HCT116
0 2.5 5 0 2.5 5
kDa
29
PCNA
HCT116
HCT116-5FUR
Metformin
ICG-001
kDa
45
OCT4
HCT116-5FUR
HCT116
Metformin
ICG-001
kDa
92
β-catenin
HCT116-5FUR
HCT116
Metformin
ICG-001
kDa
35
19
Caspase 3
Cleaved Caspase 3
HCT116-5FUR
HCT116
Metformin
ICG-001
kDa
80
CD44
HCT116-5FUR
HCT116
Metformin
ICG-001
kDa
62
AMPK
HCT116
HCT116-5FUR
Metformin
ICG-001
kDa
100
ATG9A
HCT116
HCT116-5FUR
Metformin
ICG-001
kDa
16
14
LC3B-I
LC3B-II
HCT116-5FUR
HCT116
Metformin
ICG-001
kDa
62
p-AMPK
HCT116-5FUR
HCT116
Metformin
ICG-001
kDa
45
β-actin

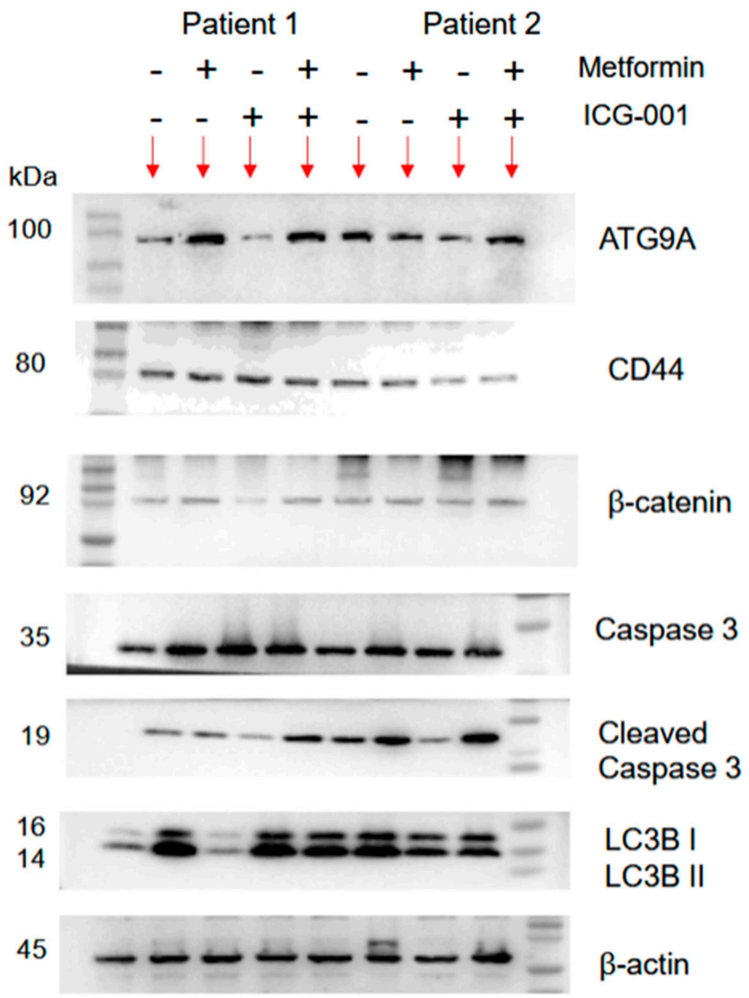

Figure S2. Original blots.

Table S1. Clinical details of the CRC patients used for tumor organoid generation.

Sl No	Patient ID	Age	Gender	Ethnicity	Location of Tumor	TNM Stage	Pathological Stage
1	Patient 1	67	Female	Caucasian	Cecum	pT4b N1b	Moderate
2	Patient 2	40	Male	Caucasian	Rectosigmoid	pT3 N0	Moderate

Table S2. List of primer sequences.

Genes	Forward Primer	Reverse Primer
β-catenin	5'-GCTTTCAGTTGAGCTGACCA-3'	5'-CAAGTCCAAGATCAGCAGTCTC-3'
CD44	5′-CGGACACCATGGACAAGTTT-3′	5′-CCGTCCGAGAGATGCTGTAG-3′
OCT4	5′-CCTGAAGCAGAAGAGGATCA-3′	5′-CCGCAGCTTACACATGTTCT-3′
Caspase 3	5′-CAAACTTTTTCAGAGGGGATCG-3′	5′-GCATACTGTTTCAGCATGGCAC-3′
LC3B	5′-CGGAAAGCAGCAGTGTACCA-3′	5′-GGCAGAAGGGAGTGTGTCTGA-3′
β-actin	5′TCACCCACACTGTGCCCATCTACGA -3′	5′- CAGCGGAACCGCTCATTGCCAATG G-3′

Table S3. IC_{50} value of metformin and ICG-001 in different CRC cells.

Drug	Cell Line	IC_{50} *
**Metformin**	HCT116	2.5 ± 0.3
	SW480	3.0 ± 0.4
	HCT116-5FUR	5.0 ± 0.5
	SW480-5FUR	7.6 ± 0.4
	SW620	7.4 ± 0.5
	RKO	8.0 ± 0.6
	HT29	5.9 ± 0.4
**ICG-001**	HCT 116	4.4 ± 0.5
	SW480	4.7 ± 0.3
	HCT 116 5FUR	6.5 ± 0.5
	SW480 5FUR	5.6 ± 0.3

Table S4. List of top 10 upregulated and downregulated genes between 5FU-resistant and parental colorectal cancer cells.

**Upregulated Genes**		**Downregulated Genes**	
Gene symbol	Log_2Fc	Gene symbol	Log_2Fc
SPARC	8.03	PSG1	−10.91
ADD2	7.16	KRT5	−9.24
OASL	6.30	TRIM29	−8.95
IFIT2	6.29	NELL2	−8.67
IFIT1	5.95	CLDN1	−8.59
CCL5	5.73	MAP7D2	−8.55
IFIT3	5.50	NRIP1	−8.32
CHRM1	5.32	CSMD3	−8.24
LONRF2	5.23	CBS	−8.19
FEV	5.10	ARHGEF38	−8.15
*Log_2Fc= 5FU-resistant vs Parental			
